# Awareness of Diabetic Retinopathy Among Known Diabetics

**DOI:** 10.7759/cureus.105168

**Published:** 2026-03-13

**Authors:** Taimoor Tahir, Muhammad Jazib Raza, Sikandar Ajmal Abbasi, Sidra Kausar, Hania Batool

**Affiliations:** 1 Medicine, Kuwait Teaching Hospital, Peshawar, PAK; 2 Surgery, Pakistan Institute of Medical Sciences, Islamabad, PAK; 3 Medicine, Pakistan Institute of Medical Sciences, Islamabad, PAK

**Keywords:** awareness, cross-sectional study, diabetic retinopathy, health literacy, ophthalmology, pakistan, patient education

## Abstract

Background and objective

Diabetic retinopathy (DR) is the leading preventable cause of irreversible blindness in working-age adults worldwide. In Pakistan, with over 30 million people living with diabetes, specific patient awareness of DR as a distinct screenable complication remains poorly studied, particularly in urban tertiary care settings. The objective of this study is to determine the prevalence of specific DR awareness among known diabetic patients at the Pakistan Institute of Medical Sciences (PIMS), Islamabad, and to identify independent sociodemographic and clinical predictors using binary logistic regression.

Methods

A cross-sectional study was conducted among 430 known diabetic patients (≥18 years) attending the outpatient departments of PIMS following IRB approval. An interviewer-administered, Urdu-translated, pilot-tested structured questionnaire was used. The primary outcome (primary outcome composite) defined specific DR awareness as correctly identifying DR, not cataract or glaucoma, as the primary ocular complication of diabetes. Bivariate associations were assessed by chi-square (Pearson or likelihood ratio as appropriate) and independent-samples t-tests. A binary logistic regression model identified independent predictors.

Results

Only 66 patients (15.3%) demonstrated specific DR awareness, despite 78.4% knowing that diabetes affects the eyes, a gap of 63.1 percentage points. Social media use was the sole independent predictor in the logistic regression (overall Wald χ² = 16.745, df = 4, P = 0.002): YouTube users OR = 3.31 (95% CI 1.44-7.62, P = 0.005); WhatsApp OR = 3.01 (95% CI 1.30-6.94, P = 0.010); and Facebook OR = 4.93 (95% CI 1.98-12.24, P = 0.001). Education showed borderline bivariate significance (likelihood ratio P = 0.047) but was not independently significant after adjustment (P = 0.477). Disease duration, gender, age, and HbA1c were all nonsignificant.

Conclusions

Specific DR awareness is critically low at 15.3%. Social media engagement is the only independent predictor identified. Targeted digital health campaigns delivering validated DR-specific education through YouTube, WhatsApp, and Facebook, combined with structured counseling at routine diabetic reviews, are urgently needed to bridge the awareness gap and prevent avoidable blindness.

## Introduction

Diabetes mellitus has become one of the defining noncommunicable diseases of the 21st century. Pakistan now ranks among the 10 most affected countries in the world, with an estimated 30 million people currently living with the condition, a figure projected to nearly double over the coming two decades [1]. The burden of diabetes is not measured in blood glucose alone; it accumulates silently in the blood vessels of the kidney, the nerve fibers of the limbs, and, perhaps most consequentially, the retinal microvasculature of the eye.

Diabetic retinopathy (DR) is the direct result of sustained hyperglycemia-mediated damage to the retinal capillary network and is the principal cause of preventable, irreversible blindness in working-age adults worldwide [2,3]. In its advanced form, DR may be complicated by diabetic macular edema (DMO), which threatens central visual acuity independently of retinopathy stage [3,4]. What makes DR particularly dangerous in resource-limited settings is its natural history: in the early and clinically significant stages, the disease is entirely asymptomatic. Visual impairment only appears when damage is far advanced and often irreversible. The window for effective intervention is therefore open during the asymptomatic phase and closes as the disease progresses.

Effective prevention depends almost entirely on patient awareness. A patient who does not know that DR exists, that it causes no early symptoms, and that an annual eye examination is mandatory regardless of visual acuity will not seek screening. Studies from comparable middle-income settings have found specific DR awareness rates of 49.5% in Jordan [5,6], 51-63% in Saudi Arabia [7,8], 34.9% in Goa [9], and 40.7% in suburban South India [10], all substantially higher than what our pilot observations suggested in the Pakistani context. Data on specific DR awareness from urban tertiary care populations in Pakistan are conspicuously absent from the published literature, with existing Pakistani studies focused primarily on general diabetes awareness in community settings [11,12] or on prevalence rates in rural cataract surgery camps [13].

This study was designed to address that gap directly. Our aims were (i) to determine the prevalence of specific DR awareness among known diabetic patients attending the outpatient departments of the Pakistan Institute of Medical Sciences (PIMS), Islamabad; (ii) to describe the broader awareness and eye care-seeking profile of this population; and (iii) to identify independent predictors of specific DR awareness using binary logistic regression.

## Materials and methods

Study design and setting

This was a descriptive, quantitative, cross-sectional study conducted at the medical and endocrinology outpatient departments of the PIMS, Islamabad. PIMS is a major national tertiary care and teaching hospital that receives patients from Islamabad, Rawalpindi, Azad Kashmir, and beyond, making it broadly representative of the urban Pakistani diabetic population seeking specialist care. Data were collected over a three-month period. Patients were attending for routine diabetes follow-up, not for ophthalmic evaluation or DR screening; this contextual distinction is important for interpreting the awareness findings.

Participants and sample size

Consecutive eligible patients presenting at the OPD during working hours were approached for participation by a trained data collector. Inclusion criteria were (i) a confirmed prior diagnosis of diabetes mellitus (Type 1 or Type 2) and (ii) age 18 years or above at the time of enrollment. Patients who were critically unwell, unable to communicate meaningfully, or had a documented preexisting nondiabetic ocular condition were excluded. One participant aged 17 years was inadvertently enrolled and excluded prior to analysis, yielding a final analytic sample of N = 430.

Sample size was calculated based on an expected specific DR awareness prevalence of approximately 30%, a 95% confidence level, and a ±5% margin of error, using the standard formula:

$n = \frac{Z^2 \cdot p (1-p)}{d^2},$

where Z is the z-score corresponding to the 95% confidence level, p is the expected prevalence (0.30), and d is the desired margin of error (0.05). This calculation yielded a minimum sample size of 323 participants. To account for a potential 10% nonresponse rate and to allow reliable subgroup analyses, the final target sample size was set at 430, which was successfully achieved over the study period.

Questionnaire and primary outcome

A structured, closed-ended questionnaire was administered face to face by a trained data collector (Appendix A, Appendix B). The questionnaire was originally developed in English, forward-translated into Urdu, and back-translated to verify conceptual equivalence. It was pilot-tested on 20 patients not included in the main study; minor wording adjustments were made before deployment. The instrument covered three domains: (i) sociodemographics and clinical characteristics (age, sex, education, diabetes duration, HbA1c, treatment modality, and frequency of physician visits); (ii) awareness and knowledge, including the primary outcome; and (iii) eye care practice and sources of health information.

The primary outcome variable, specific DR awareness (primary outcome), was operationalized as a composite variable. A participant was classified as AWARE only if they correctly identified DR (not cataract or glaucoma) as the primary ocular complication of diabetes. This definition was deliberately stringent because it reflects the specific knowledge needed to motivate appropriate screening behavior. Sixty-six patients (15.3%) met this criterion; 364 (84.7%) were classified as UN-AWARE.

The composite outcome was derived from five questionnaire items using a weighted scoring system: Question 5b (selecting Retinopathy = 2 points; more than one complication = 1 point; other responses = 0 points), Question 6 (Yes = 1 point; No = 0), Question 8 (selecting Retinopathy = 2 points; other responses = 0), Question 9 (Yes = 1 point; No = 0), and Question 10 (any frequency response = 1 point; Never = 0 points), with a maximum possible score of 7. Participants scoring 5 or above were classified as AWARE; those scoring 4 or below were classified as UN-AWARE.

Statistical analysis

All data were analyzed using IBM SPSS Statistics for Windows, Version 26.0 (Released 2018; IBM Corp., Armonk, NY, USA). Categorical variables are reported as frequencies and percentages; the continuous variable (age) is reported as mean ± SD. For bivariate associations with specific DR awareness, the Pearson chi-square was used, where all expected cell counts met the minimum threshold of 5; where one or more expected cells fell below this threshold, the likelihood ratio chi-square was applied as a more robust alternative. This applied to education level (minimum expected cell count = 3.22) and social media use, where Instagram and Twitter categories were merged prior to analysis to reduce low expected cells from 33.3% to 10% of cells below the widely accepted 20% criterion. Age was compared between the AWARE and UN-AWARE groups by an independent samples t-test after confirming equal variances (Levene’s test: F = 2.476, P = 0.116).

Binary logistic regression was performed using the Enter (simultaneous) method. The following variables were entered as predictors: age (continuous), education level, duration of diabetes, HbA1c range, social media use (merged), and gender. These were selected a priori based on clinical plausibility and the existing DR awareness literature. Model performance was evaluated using the Omnibus likelihood ratio test, the Hosmer-Lemeshow goodness-of-fit test, and Nagelkerke R². Statistical significance was defined as P < 0.05 throughout.

Ethical considerations

Ethical approval was granted by the Institutional Review Board (IRB) of the Prime Foundation Pakistan, Peshawar, on May 31, 2023. The IRB specifically approved verbal informed consent in lieu of written consent, given the noninterventional, observational nature of the study and the significant proportion of participants with limited literacy, for whom written documentation could create unnecessary barriers. All participants were informed of the study purpose, the voluntary nature of participation, and their right to withdraw at any time without consequence. No personally identifiable information was recorded, and all data were handled in strict confidence.

## Results

A total of 430 known diabetic patients completed the study. The sample comprised 225 males (52.3%) and 205 females (47.7%), with a mean age of 49.5 ± 12.3 years. Educational attainment was generally low: 207 participants (48.1%) had not completed matric-level schooling, and only 21 (4.9%) held a postgraduate qualification. The majority had been living with diabetes for five years or less (208, 48.4%), while only 14 (3.3%) reported a duration of more than 20 years. Most participants were managed with oral hypoglycemic agents (266, 61.9%), and 289 (67.2%) reported HbA1c values above 7%, indicating suboptimal glycemic control in approximately two-thirds of the cohort. Full sociodemographic and clinical details are presented in Table 1.

**Table 1:** Sociodemographic and clinical characteristics of study participants (N = 430) Gender sub-counts for categorical variables are estimated proportionally; total column figures are taken directly from IBM SPSS Statistics for Windows, Version 26.0 (Released 2018; IBM Corp., Armonk, NY, USA) frequency output.

Variable	Male, n = 225 (52.3%)	Female, n = 205 (47.7%)	Total, N = 430
Age, mean ± SD (years)	48.9 ± 10.1	50.2 ± 13.7	49.5 ± 12.3
Education level			
Below matric	105 (50.7%)	102 (49.3%)	207 (48.1%)
Matric	30 (55.6%)	24 (44.4%)	54 (12.6%)
Intermediate	38 (52.1%)	35 (47.9%)	73 (17.0%)
Bachelors	38 (50.7%)	37 (49.3%)	75 (17.4%)
Masters and above	14 (66.7%)	7 (33.3%)	21 (4.9%)
Duration of diabetes			
≤5 years	103 (49.5%)	105 (50.5%)	208 (48.4%)
≤10 years	86 (53.8%)	74 (46.3%)	160 (37.2%)
≤20 years	24 (50.0%)	24 (50.0%)	48 (11.2%)
>20 years	12 (85.7%)	2 (14.3%)	14 (3.3%)
Method of diabetes management			
Tablets only	143 (53.8%)	123 (46.2%)	266 (61.9%)
Insulin only	27 (52.9%)	24 (47.1%)	51 (11.9%)
Injectables + tablets	51 (49.5%)	52 (50.5%)	103 (24.0%)
Lifestyle modification	4 (40.0%)	6 (60.0%)	10 (2.3%)
HbA1c status			
HbA1c >7% (suboptimal control)	154 (53.3%)	135 (46.7%)	289 (67.2%)
HbA1c ≤7% (at target)	55 (50.9%)	53 (49.1%)	108 (25.1%)
Not checked	16 (48.5%)	17 (51.5%)	33 (7.7%)

**Figure 1:**
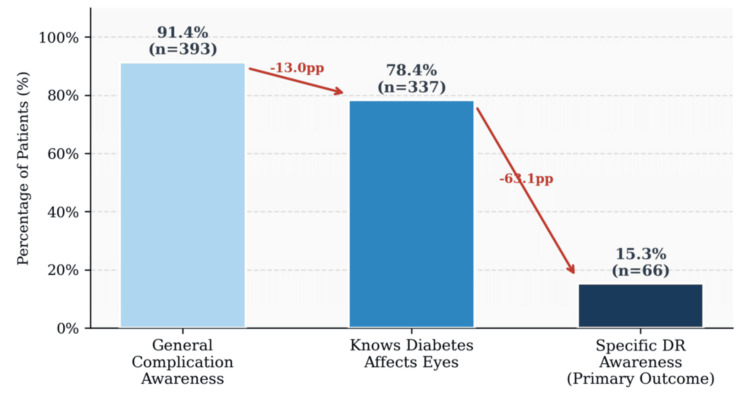
Awareness cascade from general diabetes awareness to specific DR awareness (N = 430) Values represent the percentage of total participants. DR, diabetic retinopathy

The full awareness and eye care profile is presented in Table 2. The awareness cascade, from general to specific knowledge, is visually summarized in Figure 1. While 91.4% of participants were aware of at least one complication of diabetes and 78.4% knew that diabetes can affect the eyes, only 66 participants (15.3%) correctly identified DR as the primary ocular complication and were classified as specifically AWARE. This represents a 63.1-percentage-point drop from general ocular awareness to specific DR awareness and constitutes the most clinically important finding of this study.

**Table 2:** Awareness, knowledge, and eye care profile (N = 430) Primary outcome composite: AWARE = correctly named DR (not cataract or glaucoma) as the primary ocular complication. A total of 13% of participants incorrectly identified cataract as the primary complication. DR, diabetic retinopathy

Variable	n	%
General awareness of at least one diabetes complication		
Yes	393	91.4
No	37	8.6
Aware that diabetes can affect the eyes		
Yes	337	78.4
No	93	21.6
Specific DR awareness - primary outcome (composite)		
AWARE: correctly identified DR as the primary ocular complication	66	15.3
UN-AWARE	364	84.7
Ever had an eye examination since diabetes diagnosis		
Yes	274	63.7
No - never examined since diagnosis	156	36.3
Frequency of eye examinations		
Never	212	49.3
Yearly	136	31.6
Every six months	46	10.7
Every three months	25	5.8
Monthly	11	2.6
Primary source of information about eye complications of diabetes		
General practitioner	183	42.6
Not known/never informed	99	23.0
Endocrinologist	79	18.4
More than one source	48	11.2
Nonsocial media (pamphlets, etc.)	15	3.5
Ophthalmologist	6	1.4
Specific eye complications named by participants		
None named/did not know	97	22.6
Cataract only	56	13.0
Glaucoma only	30	7.0
DR only	89	20.7
More than one complication named	158	36.7

Among the remaining participants, 13% incorrectly identified cataract as the primary ocular complication of diabetes, and 22.6% were unable to name any eye complication at all. Regarding eye care practices, 274 participants (63.7%) had undergone at least one eye examination since their diabetes diagnosis, but 212 (49.3%) reported never having had a routine eye check-up when asked about frequency. Only 136 (31.6%) reported annual eye examinations, which is the minimum recommended interval for diabetic patients with no existing retinopathy. The most frequently reported source of information about eye complications was the general practitioner (183, 42.6%), while only six patients (1.4%) cited an ophthalmologist as their primary information source.

**Figure 2:**
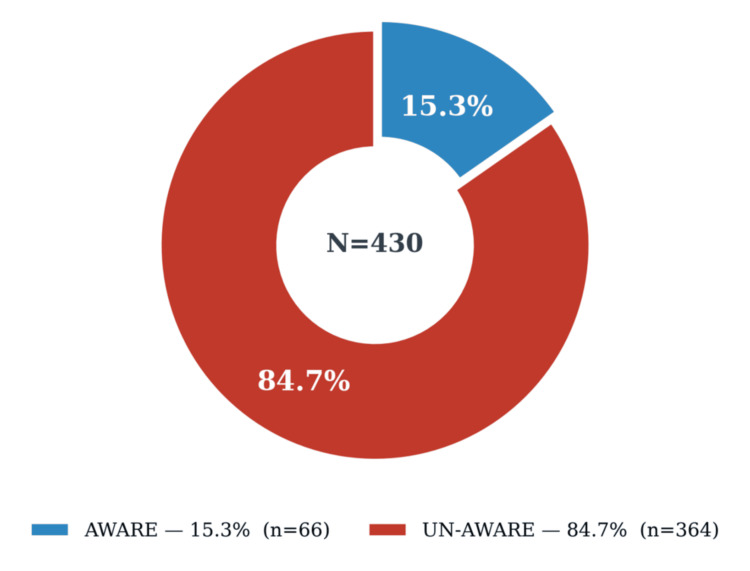
Specific DR awareness among known diabetic patients (N = 430) Only 15.3% (n = 66) were AWARE; 84.7% (n = 364) were UN-AWARE. Primary outcome: composite variable. DR, diabetic retinopathy

Full bivariate results are presented in Table 3 and Figure 2. Age was similar between the AWARE and UN-AWARE groups (48.9 ± 10.3 vs 49.7 ± 12.6 years; t = -0.479, df = 428, P = 0.632). Education level showed a borderline association with specific DR awareness on the likelihood ratio test (LR χ² = 9.651, df = 4, P = 0.047), although the Pearson chi-square did not reach significance (χ² = 8.691, P = 0.069). The likelihood ratio test was appropriate given the minimum expected cell count of 3.22. There was no clear educational gradient: below matric participants accounted for the largest number of AWARE patients (n = 41) by virtue of group size rather than a higher proportional awareness rate.

**Table 3:** Bivariate analysis: associations with specific DR awareness (N = 430) ^*^ P < 0.05 LR = likelihood ratio χ² (used where ≥1 expected cell count was <5: education min expected = 3.22; social media after merging Instagram + Twitter min expected = 3.08). T-test = independent-samples t-test (Levene P = 0.116, equal variances assumed). DR, diabetic retinopathy

Predictor variable	AWARE (n = 66)	UN-AWARE (n = 364)	Test	Statistic (df)	P-value
Age (years), mean ± SD	48.9 ± 10.3	49.7 ± 12.6	t-test	t = -0.479 (428)	0.632
Education level - overall			LR χ²	9.651 (4)	0.047^*^
Below matric	41 (62.1%)	166 (45.6%)			
Matric	3 (4.5%)	51 (14.0%)			
Intermediate	8 (12.1%)	65 (17.9%)			
Bachelors	10 (15.2%)	65 (17.9%)			
Masters and above	4 (6.1%)	17 (4.7%)			
Duration of diabetes - overall			χ²	1.168 (3)	0.761
≤5 years	32 (48.5%)	176 (48.4%)			
≤10 years	24 (36.4%)	136 (37.4%)			
≤20 years	9 (13.6%)	39 (10.7%)			
>20 years	1 (1.5%)	13 (3.6%)			
Social media use - overall			LR χ²	23.807 (4)	<0.001^*^
None	31 (47.0%)	72 (19.8%)			
YouTube	13 (19.7%)	89 (24.5%)			
WhatsApp	12 (18.2%)	76 (20.9%)			
Instagram/Twitter (merged)	1 (1.5%)	25 (6.9%)			
Facebook	9 (13.6%)	102 (28.0%)			
Gender (male/female)	225/205			χ² = 0.897 (1)	0.344
Frequency of doctor visits			χ²	1.515 (3)	0.679

Social media use showed the strongest bivariate association (LR χ² = 23.807, df = 4, P < 0.001; Pearson χ² = 25.100, P < 0.001). It may appear counterintuitive that patients reporting no social media use had the highest raw awareness count (n = 31, 30.1% within the group); this apparent paradox arises because the None group was the largest absolute group and includes patients who may access health information through other channels. This pattern is fully explained by the multivariate logistic regression below, which adjusts for all confounders simultaneously. Disease duration (χ² = 1.168, P = 0.761), gender (χ² = 0.897, P = 0.344), and frequency of doctor visits (χ² = 1.515, P = 0.679) were all nonsignificant.

**Figure 3:**
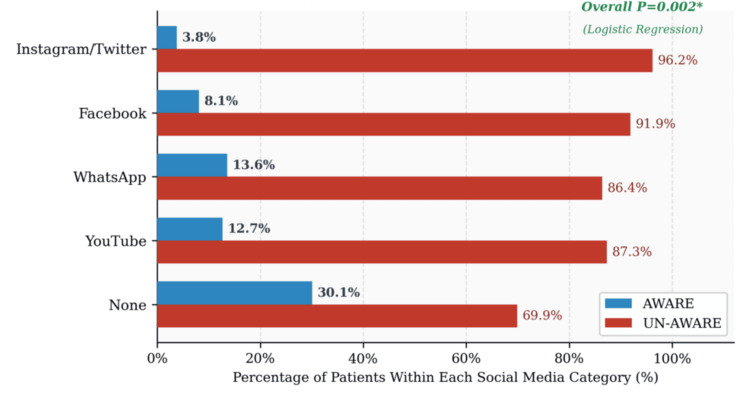
Specific DR awareness by primary social media platform used (N = 430) Bars show the proportion AWARE (blue) and UN-AWARE (red) within each platform category. Overall likelihood ratio P < 0.001; logistic regression overall P = 0.002. DR, diabetic retinopathy

The logistic regression model achieved overall statistical significance (Omnibus χ² = 31.572, df = 15, P = 0.007) and demonstrated adequate calibration (Hosmer-Lemeshow χ² = 5.322, df = 8, P = 0.723). Nagelkerke R² was 0.123, indicating that the model accounts for approximately 12.3% of the variance in specific DR awareness. Full results are presented in Table 4 and visualized in Figure 3.

**Table 4:** Binary logistic regression: independent predictors of specific DR awareness (N = 430) ^*^ P < 0.05 ^†^ Instagram and Twitter merged (n = 26 total, only one AWARE patient) B = regression coefficient; OR = 14.263 is statistically unstable (95% CI 1.659-122.585) and should not be interpreted as a robust estimate. Model fit: Omnibus χ² = 31.572, df = 15, P = 0.007. Hosmer-Lemeshow P = 0.723 (adequate calibration). Nagelkerke R² = 0.123. Classification note: Due to class imbalance (15.3% AWARE), the model classifies all cases as UN-AWARE at the default cutoff (sensitivity = 0%, specificity = 100%, overall accuracy = 84.7%). This model is reported for predictor identification, not for clinical classification. DR, diabetic retinopathy

Variable (reference)	B	SE	Wald χ² (df)	P	OR	95% CI (lower)	95% CI (upper)
Age (years, continuous)	0.015	0.014	1.162 (1)	0.281	1.015	0.988	1.042
Education level (ref: below matric)			3.503 (4)	0.477			
Matric	0.892	0.649	1.888 (1)	0.169	2.440	0.684	8.708
Intermediate	0.228	0.449	0.257 (1)	0.612	1.256	0.521	3.029
Bachelors	−0.175	0.440	0.158 (1)	0.691	0.839	0.354	1.989
Masters and above	−0.501	0.632	0.627 (1)	0.429	0.606	0.176	2.093
Duration of diabetes (ref: ≤5 years)			0.778 (3)	0.855			
≤10 years	0.099	0.338	0.085 (1)	0.770	1.104	0.569	2.143
≤20 years	−0.014	0.474	0.001 (1)	0.976	0.986	0.389	2.497
>20 years	0.948	1.127	0.708 (1)	0.400	2.581	0.284	23.481
Social media use (ref: none) - only significant predictor			16.745 (4)	0.002^*^			
YouTube	1.196	0.426	7.892 (1)	0.005^*^	3.306	1.435	7.615
WhatsApp	1.101	0.427	6.652 (1)	0.010^*^	3.006	1.302	6.938
Instagram/Twitter^†^	2.658	1.098	5.863 (1)	0.015^*^	14.263	1.659	122.585
Facebook	1.594	0.465	11.774 (1)	0.001^*^	4.925	1.981	12.242
Gender (female vs male)	0.008	0.292	0.001 (1)	0.978	1.008	0.569	1.786

**Figure 4:**
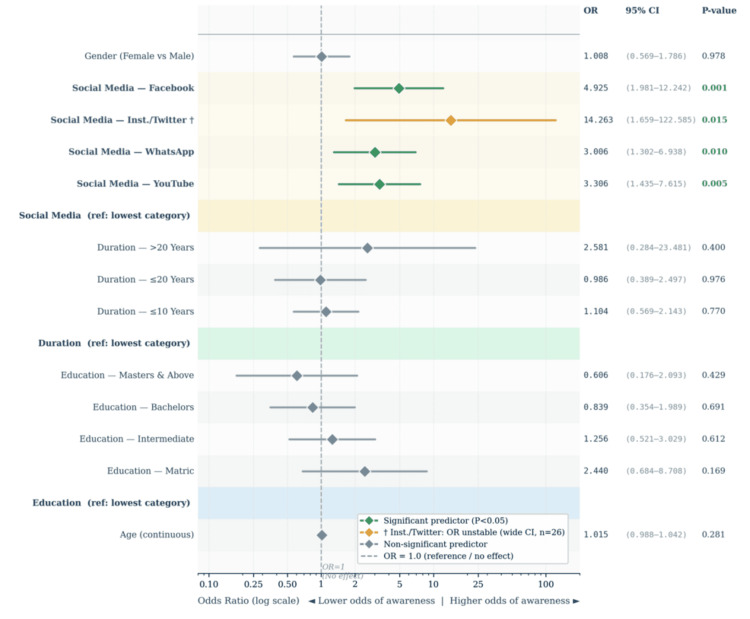
Forest plot: all predictors of specific DR awareness (binary logistic regression, N = 430) Each row represents one predictor sub-category. Diamond = adjusted OR; line = 95% CI on log scale. Dashed line = OR of 1.0 (null). Green = significant (P < 0.05); grey = nonsignificant; amber (^†^) = statistically unstable. Social media use was the only independently significant predictor. DR, diabetic retinopathy

Social media use was the only variable that reached independent statistical significance after simultaneous adjustment for all other predictors (overall Wald χ² = 16.745, df = 4, P = 0.002). Compared with patients who used no social media, Facebook users had nearly five times the odds of being specifically aware of DR (OR = 4.925, 95% CI 1.981-12.242, P = 0.001). YouTube users were over three times as likely to be aware (OR = 3.306, 95% CI 1.435-7.615, P = 0.005), and WhatsApp users similarly so (OR = 3.006, 95% CI 1.302-6.938, P = 0.010). The merged Instagram/Twitter category also reached significance (OR = 14.263, P = 0.015) but carries a very wide confidence interval (95% CI 1.659-122.585), reflecting severe instability due to only one AWARE patient among 26 users in this group. This estimate is flagged with a dagger (^†^) in both Table 4 and Figure 4 and must not be interpreted as a reliable effect size.

After adjusting for social media use, education level was no longer independently significant (overall Wald P = 0.477). Disease duration (overall Wald P = 0.855), gender (Wald P = 0.978), age (Wald P = 0.281), and HbA1c were all nonsignificant in the adjusted model. It is important to note that the severe class imbalance in the outcome (15.3% AWARE vs 84.7% Un-AWARE) causes the model to classify every patient as UN-AWARE at the default 0.5 probability threshold (sensitivity = 0%, specificity = 100%). The model is reported for predictor-identification purposes, not for individual-level classification.

## Discussion

The central finding of this study is both simple and alarming: despite attending a major national tertiary care hospital with an established diabetes diagnosis, only 15.3% of patients in this cohort had specific awareness of DR as the primary ocular complication of their disease. This figure is substantially lower than equivalent rates reported from Jordan (49.5%) [5], Saudi Arabia (51-63%) [7,8], and India (34.9-40.7%) [9,10]. It is rendered more troubling by the context: 78.4% of the same patients already knew that diabetes can affect the eyes. The 63.1 percentage-point gap between general ocular awareness and specific DR awareness is not a trivial statistical artifact; it represents a failure to communicate the most clinically critical piece of information to patients at every point at which they interact with the healthcare system.

This distinction between general and specific awareness matters precisely because it determines behavior. A patient who knows that “diabetes affects the eyes” but associates this with cataract, as 13% of our cohort did, will wait for blurred vision before seeking an ophthalmologist. DR, however, causes no early visual symptoms; by the time symptoms appear, proliferative disease or advanced DMO may already be present, and the window for straightforward photocoagulation or intravitreal therapy to prevent blindness may have closed [2,3]. The cost of this knowledge gap, measured in preventable sight loss, is significant.

The logistic regression identified social media use as the only independent predictor of specific DR awareness, with Facebook users being nearly five times more likely to be aware than nonusers, and YouTube and WhatsApp users approximately three times more likely. These associations held after simultaneously controlling for age, education, disease duration, HbA1c, and gender, confirming that digital engagement is not simply a proxy for higher socioeconomic status or formal education but an independent driver of specific health knowledge. This finding aligns with Fallatah’s cross-sectional study in Jeddah, which identified social media as a significant information source for DR knowledge [8], and with Abdu et al.’s findings that digital media access predicted better DR awareness in a Saudi awareness camp setting [7]. In Pakistan, where diabetes clinics are frequently overcrowded and physician contact time per patient is severely limited, social media represents a uniquely scalable and cost-effective platform for delivering targeted, validated DR education to a population that is already online.

The attenuation of the education effect from borderline significance in bivariate analysis to nonsignificance in the adjusted model is noteworthy. One plausible explanation is partial mediation: better-educated patients may be more likely to engage actively with health content on digital platforms, and it is this downstream digital engagement, rather than formal education per se, that drives specific DR knowledge acquisition. This hypothesis is plausible but cannot be confirmed from a cross-sectional design; longitudinal research with mediation analysis would be needed to establish the causal pathway.

The absence of a significant association between doctor visit frequency and specific DR awareness warrants careful interpretation. It would be premature to conclude that clinical contact is irrelevant to DR education. A more measured reading is that, within this cohort, the routine outpatient consultation, typically focused on glycemic monitoring and medication adjustment under time pressure, does not reliably deliver specific and granular DR knowledge. This finding, echoed in Turkish diabetic clinic populations [11], does not argue against using the clinic as a venue for education but rather emphasizes that education must be active and structured, not incidental. Standardized DR counseling protocols with trained support staff, validated visual aids in Urdu, and mandatory referral checklists for ophthalmologic review are practical interventions that could be implemented without restructuring the healthcare system.

Strengths and limitations

This study has several methodological strengths. The sample size of 430 exceeds the calculated minimum; the questionnaire was rigorously translated and pilot-tested, and the interviewer-administered format controlled for literacy-related response bias. The use of binary logistic regression to identify independent predictors rather than relying on bivariate associations alone represents a meaningful analytical advance over many published DR awareness studies from comparable settings.

Several limitations must be acknowledged openly. First, the cross-sectional design precludes any causal interpretation; all associations described here are observational. Second, convenience sampling at a single urban tertiary center limits generalizability to rural, primary care, and community diabetic populations, where awareness may be even lower. Third, severe class imbalance in the outcome variable (15.3% AWARE) means the logistic regression model has zero sensitivity at the default threshold and should not be used for individual prediction. Fourth, the OR = 14.263 for the merged Instagram/Twitter category is statistically unstable (95% CI 1.659-122.585) and must not be interpreted as a robust effect estimate. Fifth, self-reported HbA1c values are susceptible to recall and social desirability bias. Sixth, the events-per-variable ratio in the regression was approximately 4.4 (66 events across 15 predictors), which is below the conventionally recommended threshold of 10; results should be interpreted with appropriate caution. Finally, while verbal consent was specifically approved by the IRB, the absence of written consent documentation may be considered a limitation by some readers.

## Conclusions

Known diabetic patients attending a major Pakistani tertiary care hospital demonstrate critically low specific awareness of DR at 15.3%, with a 63.1 percentage-point gap between those who know that diabetes can affect the eyes and those who correctly understand what DR actually is. More than one-third of participants had never undergone a single eye examination since their diabetes diagnosis, despite the availability of effective and inexpensive screening. Social media use emerged as the only independent predictor of specific DR awareness, with Facebook, YouTube, and WhatsApp users being three to five times more likely to have this knowledge than nonusers after full multivariate adjustment. These findings convey two clear and actionable messages. From a public health perspective, validated DR-specific educational content must be actively disseminated through the digital platforms that Pakistani diabetic patients already use daily. From a clinical standpoint, every routine diabetic consultation should include a brief, structured discussion about DR: what it is, the fact that it causes no early warning symptoms, and the necessity of an annual eye examination. Both interventions are low cost and do not require additional infrastructure. The cost of failing to implement them is preventable blindness. Future research should prioritize multicenter prospective studies with random sampling to generate generalizable prevalence estimates, mediation analyses to clarify the social media-awareness pathway, and intervention studies to evaluate the effectiveness of structured digital DR education campaigns in Pakistani settings.

## Appendices

Appendix A: Study questionnaire (English)

Subject no: _________

Disclaimer: The information gathered by this research tool is just for educational purposes; your information won't be given/disclosed to a third party without your written consent.

Gender: ____________                                                             Education: _________________

Age: _______________                                                              Address/City: ______________   

1. How long have you been diabetic?

A. Less than 5 years B. 5 years C. 10 years D. 20 years E. More than 20 years

2. How do you control your diabetes?

A. Injection (insulin) B. Tablets (oral) C. Injection and oral D. Others (exercise, dietary control)

3. Have you ever checked your HBA1C (Hemoglobin A1 test)?

Yes / No

· If yes - Range   Above 7 / Below 7

4. How frequently do you go to your doctor to check for diabetic control?

  A. 1 month B.  3 months C. 6 months D.  Irregularly

5. Are you aware of the complications of diabetes?

Yes / No

· If yes, which one: ischemic heart disease / Nephropathy (hypertension) / Diabetic foot / Retinopathy

6.  Do you know that the eye is also affected by diabetes?

Yes / No

· If yes, who were you informed by: General Physician / Endocrinologist / Ophthalmologist / Media

7. What form of social media do you use most frequently?

A. YouTube B. WhatsApp C. Instagram D. Facebook E. Twitter

8. Which of the diabetic eye complications are you familiar with?

A.  Cataract B.  Glaucoma C.  Retinopathies

9. Are you aware that an eye checkup is mandatory, even if your diabetes is in control?

Yes / No

10.  How frequently do you visit your doctor for an eye checkup?

A. 1 month B.  3 months C.  6 months D.  Yearly E.  Never did that

Level of awareness:

Question 5b

4 = 2, 5 = 1

1-3 = 0

Question 6

Yes = 1

No = 0

Question 8

3 = 2

1-2 = 0

Question 9

Yes = 1

No = 0

Question 10

1-4 = 1

5 = 0

Level

5 or more = Aware

4 or less = Unaware

Appendix B: Study questionnaire (Urdu translation)

موضوع نمبر: _____

دستبرداری

اس تحقیقی آلے کے ذریعہ جمع کردہ معلومات صرف تعلیمی بنیاد کے لئے ہیں۔ آپ کی معلومات آپ کی تحریری رضامندی کے بغیر کسی تیسرے فریق کو نہیں دی جائے گی / ظاہر نہیں کی جائے گی.

  جنس: ________     تعلیم________

  عمر: _______ ایڈریس/شہر: ____   

1.  آپ کتنے عرصے سے ذیابیطس کے مریض ہیں؟

           A (  5 سال  (B                  10 سال  (C               20 سال (D           20 سال سے زیادہ

2.  ذیابیطس کو کیسے کنٹرول کریں؟

 (A               انجکشن (انسولین)  ( B             گولیاں (منہ سے کھانا والی)  ( C          انجکشن اور گولیاں ۔( D          دوسرے (ورزش، غذائی کنٹرول)

3.  کیا آپ نے کبھی اپنے ایچ بی اے 1 سی (ہیموگلوبن اے 1 ٹیسٹ) کی جانچ کی ہے؟

          ہاں / نہیں

· اگر ہاں - رینج     7 سے اوپ / 7 سے نیچے

4.  ذیابیطس پر قابو پانے کے لئے آپ کتنے وقت کے بعد اپنے ڈاکٹر کے پاس جاتے ہیں؟

(A   1 ماہ. (B                3 ماہ(C                      6 ماہ.    ( D            بے قاعدگی سے

5. کیا آپ ذیابیطس کی پیچیدگیوں سے آگاہ ہیں؟

           ہاں / نہیں

· اگر ہاں - کونسی:         اسکیمک دل کی بیماری /        نیفروپیتھی (ہائی بلڈ پریشر)     /          ذیابیطس پاؤں        /         ریٹینوپیتھی

6.  کیا آپ جانتے ہیں کہ آنکھ بھی ذیابیطس سے متاثر ہوتی ہے؟

                    ہاں / نہیں

· اگر ہاں - آپ کو کس نے مطلع کیا تھا:       جنرل فزیشن /    اینڈوکرائنولوجسٹ /      آنکھوں کے ماہر /       میڈیا

7.  آپ کس قسم کا سوشل میڈیا سب سے زیادہ استعمال کرتے ہیں؟

 (AیوٹیوبB           (واٹس ایپ ( C        انسٹاگرام.  (D        فیس بک (E               . ٹویٹر

8.  ذیابیطس کی آنکھوں کی پیچیدگیوں میں سے کس کے بارے میں آپ جانتے ہیں؟

(A موتیا.B                  ( گلوکوما سی.       (Cریٹینوپیتھیز

9. کیا آپ جانتے ہیں کہ آنکھوں کا چیک اپ لازمی ہے، بھلے ہی آپ کی ذیابیطس کنٹرول میں ہو؟

            ہاں / نہیں

10.  آپ آنکھوں کے چیک اپ کے لئے کتنی بار اپنے ڈاکٹر کے پاس جاتے ہیں؟

(A   1 ماہ( B               3 ماہ (C                   6 ماہ.               ( DسالانہE                  ( ان میں سے کوئی نہیں
